# Exploring perceptions of common practices immediately following burn injuries in rural communities of Bangladesh

**DOI:** 10.1186/s12913-018-3287-3

**Published:** 2018-06-18

**Authors:** Animesh Biswas, Abu Sayeed Md Abdullah, Koustuv Dalal, Toity Deave, Fazlur Rahman, Saidur Rahman Mashreky

**Affiliations:** 1 Reproductive and Child Health Department, Centre for Injury Prevention and Research, Bangladesh (CIPRB), House B 162, Road 23, New DOHS, Mohakhali, Dhaka, 1206 Bangladesh; 2 Department of Public Health Science, School of Health and Medical Sciences, Örebro University, Örebro, Sweden; 3 Centre for Child & Adolescent Health, Faculty of Health & Applied Sciences, University of the West of England, Bristol, UK; 4 Bangladesh University of Health sciences (BUHS), Dhaka, Bangladesh

**Keywords:** Perceptions, Rural community, Burn injury, Bangladesh

## Abstract

**Background:**

Burns can be the most devastating injuries in the world, they constitute a global public health problem and cause widespread public health concern. Every year in Bangladesh more than 365,000 people are injured by electrical, thermal and other causes of burn injuries. Among them 27,000 need hospital admission and over 5600 people die. Immediate treatment and medication has been found to be significant in the success of recovering from a burn. However, common practices used in the treatment of burn injuries in the community is not well documented in Bangladesh. This study was designed to explore the perception of local communities in Bangladesh the common practices used and health-seeking behaviors sought immediately after a burn injury has occurred.

**Methods:**

A qualitative study was conducted using Focus Group Discussions (FGD) as the data collection method. Six unions of three districts in rural Bangladesh were randomly selected and FGDs were conducted in these districts with six burn survivors and their relatives and neighbours. Data were analyzed manually, codes were identified and the grouped into themes.

**Results:**

The participants stated that burn injuries are common during the winter in Bangladesh. Inhabitants in the rural areas said that it was common practice, and correct, to apply the following to the injured area immediately after a burn: egg albumin, salty water, toothpaste, kerosene, coconut oil, cow dung or soil. Some also believed that applying water is harmful to a burn injury. Most participants did not know about any referral system for burn patients. They expressed their dissatisfaction about the lack of available health service facilities at the recommended health care centers at both the district level and above.

**Conclusions:**

In rural Bangladesh, the current first-aid practices for burn injuries are incorrect; there is a widely held belief that using water on burns is harmful.

## Background

Burns are a major public health problem which ranks fourth among all injuries worldwide [1]. More than 300,000 people die every year due to a burn injury, 90% of them occur in low and middle income countries [1–3]. The National Health and Injury Survey data in Bangladesh found that around 365,000 people experience burn injuries every year from electrical, thermal and other causes of burns. Of these 27,000 needed hospital admission and over 5600 people died [4]. Mortality rates from burn injuries are higher among rural women than men and these are mostly unintentional in nature. The majority of burn injuries occur in the home with the kitchen being the most common place where a burn will take place [4, 5]. Burn injuries require long hospital stays and are a major cause of disability [5], they also lead to a huge economic and social burden on the families involved [6, 7]. The cost of burn treatment is high and burns require dedicated treatment settings with specialist doctors and equipment; these are not always available in many low income countries [7–9]. The first hour following a burn is called the ‘golden hour’; appropriate management within this time can save lives and reduce its severity and minimizes the duration of hospital stay [10]. The history of burn first-aid shows a range of treatments that have little or no evidence to support the use of many of them. While most regulatory bodies currently recommend applying cold water for 20-min, others state that cold water can still be effective if applied up to 3 h after the burn is sustained [11]. The prevention and management of burn injuries are still not conducted efficiently in low- and middle-income countries, like Bangladesh [12]. This is specifically the case in rural areas where burn patients are usually treated in the community by unqualified service providers [13] rather attending a health facility. Primary health care centers, called ‘upazila health complexes’ are not equipped to manage burn patients and there are few places where doctors are trained on the emergency management of severe burns (EMSB) and providing care at primary health centers [14]. Awareness of first-aid protocols for both burn injuries and the improvement of acute burn care management is essential for the reduction of death and disability due to burn injury in Bangladesh [15].

This study explored the common first-aid practices that are used immediately following a burn injury in rural communities in Bangladesh. This study also identified the consequences of immediate management after burn injury and the factors affecting to choose the health care providers.

## Methods

A qualitative study design was adopted during January to April 2015 where focus group discussion (FGDs) were used as the data collection technique.

### Study place and population

The study was conducted in Narail, Bagerhat and Netrakona districts of Bangladesh. Three districts in Bangladesh were selected to take part by purposively. Within each district, two sub-districts (uapzilas) were randomly selected using random sampling. The population of the uapzilas varied between 200,000 to 400,000 residents. From each of the uapzilas, one union (a small unit of an upazila, which consists of a population size of around 12,000 to 20,000 people) was randomly selected for study inclusion. Residents in these unions who were over 18 years of age and resided in the villages of the burn survivors were invited to take part in the study.

### Sample size

A total of six focus groups (FGDs) were conducted, one in each union in each upazilas. In each union, one moderate to severe burn case was identified from the upazilas health centers’ previous year admission record. If a union had more than one burn survivor, one patient was selected randomly. The age range of burn survivors was 10 months to 18 years (Table 1).

**Table 1:** Burn survivour’s characteristics

Case	Areas	Age (in years/ months)	Case presentation
Case 1	Mollarhat upazila, Bagerhat district	10 months	Put the hands in the hot cooked rice dish.
Case 2	Fakirhat upazila, Bagerhat District	01 year	Put the lower limb into the oven when mother was cooking.
Case 3	Durgapur upazila, Netrakona district	18 years	Suicidal attempt.
Case 4	Purbadhala upazila, Netrakona district	5 years	During carrying traditional lamp (Kupibati) at evening inside the home, fire caught into cloth.
Case 5	Sadar upazila, Narail District	7 years	Put oil in the fire when playing and caught fire.
Case	Lohagora upazila, Narail District	18 years	Fire caught from short circuit from electric cable.

The FGDs were held in the villages where a burn survivor had resident and took place within the courtyards of the villages, close to where the burn survivor lived. Villagers who were relatives or were known to the burn survivor were invited to participate in the FGDs through the support of government health care providers who were working in those communities. Between nine and eleven participants took part in each FGD; a total of 61 participants with an age range of 18 to 70 years (mean age = 44 years). Participants included school teachers, religious leaders, housewives, parents, community leaders, elected local government members, village doctors and medicine shop keepers (Table 2).

**Table 2:** Focus-group discussion schedule

Areas of discussion	Types of prompts used
Perception of burn injuries and primary first aid in the community.	General views of burn injuries in rural communities, Common causes of burn in the community. Ideas about immediate care (first aid) following a burn injury. Where the community obtain the information about what they need to do for treating a burn injury, (the source of information)
Community practices immediately after a burn injury occurs.	What are the practices in the community immediately after a burn injury occurs? What treatment(s) is (are) applied to a burn patient? Where do rural communities go if any complications arise from the burn injury? From whom do rural communities usually seek health care? What are the challenges faced by rural communities to offer first aid and ensure appropriate referral of the patient?

### Data collection

Two trained field research officers conducted the FGDs using a schedule with a number of prompts (Table 1). One acted as facilitator for the group and the other took note. The FGD schedule was developed by a number of consultative meetings and finalized following field testing. Each FGD was audio-recorded and key notes were taken. Informed, written consent was provided by all participants prior to starting data collection.

### Data processing and analysis

From the audio-recordings and handwritten notes, transcripts were prepared by the two research officers. One quarter of the transcripts were checked by the research officers to ensure quality of the data as well as consistency with the audio tape. The transcripts were then translated from Bengali to English by the research officers. They then read through and listened to the transcripts and identified open codes from the data [16]. Selective coding of the data was then undertaken and these codes were grouped into categories under each theme [17].

## Results

The results of the focus group discussions are presented and described by theme that emerged from the data.

Participants reported that, in rural Bangladesh, burn injuries generally occur during the dry and predominantly during the winter season. Many misperceptions and malpractices persist about the type of first-aid needed following a burn injury. The majority of the participants did not have sufficient knowledge and understanding about the correct treatment, care and prevention of burn injuries.

### Perception regarding causes of burn injury at community

The FGD participants identified the following as the most common causes of burn injuries: lack of awareness of a potential hazard when using the oven; not stopping the fire once cooking has finished which needs to be put out; the use of a firelight to help movement from one place to another place at night and, upon arrival, forgetting to put out the fire rather than throw it somewhere else, where it may catch alight; using kupibati (kerosene lamp); having mosquito coils lit at night; negligence in handling hot water, hot rice or hot curry; carelessness during cooking and, some participants mentioned, faulty electric wiring. A woman aged 21 years with a burn injury reported:

*“I was making tea in the oven in the kitchen; my eight months’ daughter was crying in the cart, I suddenly rushed to catch my daughter. Part of my clothes caught fire and I did not see that at first, later when it started to spread, neighbors saw me and threw water [over woman].”*

One of the older male participants mentioned:

*“Children are at high risk. We don’t put water [on the fire] at the end of cooking in the yard. Children crawl in the yard and put their hands inside the oven which causes burn. We found at least five recently delivered mothers who caught fire in last one year when they sit with their newborn in front of the flame to get warm.”*

Another woman said *“ we do make mistakes and we understand after any accident occur, we never careful about the fire around the oven which is the common place to get burned in villages.”*

### Perception about severity and outcome of burn injuries

Rural communities have a poor understanding of the severity and effect of a burn injury. Most of the participants thought that burns occur often, that they can be easily managed at home, that burns only cause peripheral skin damage and that, once the skin starts to shrink, the burn will heal. They knew that burns cause blisters, irritation, burning sensation, pus formation, pain and fever but they thought that the traditional healer (kabiraj) and village doctors could treat any burn. There were three different perspectives about cooking over open flames and these are described below. One of the older male participants who was a farmer described the danger of large cooking pots and cooking:

*“I have seen one of my relatives burnt on the face, she fell into hot water whilst taking something out from the oven. Neighbors said that she will not survive; I cannot even recognize her face. We bought her to our kabiraj, he treated for a month and my relative is now quite ok. We did not go to the hospital”.*

Another participant explained the threat that flames have when cooking on open fires and the importance of timely referral to the appropriate healthcare facility:

*“One of my neighbors was burned by flames from the oven in the kitchen. We noticed that the house was burning; we threw lots of water [on it]. The woman was badly injured; a health worker told us the patient will not survive if we delayed the transfer. We understood and immediately sent the woman to the district facility.”*

One relative of a burn survivor highlighted the importance of supervision of young children when they were in the proximity of the kitchen area:

*“Last winter I was preparing food in the kitchen and after I finished cooking, I forgot to put water in the oven [that was] in the yard. My one-year-old son crawled to the oven and put his legs inside the oven and was severely burnt”.*

### Practices of the community after a burn injury occurs

Inhabitants in the communities in Bangladesh practice a number of incorrect first-aid treatments following a burn injury and this has been taking place for many years. In the majority cases, the participants of the study didn’t see that there was a need to take a burn survivor to a hospital facility, instead they managed it locally, mostly by using traditional healers or a ‘kabiraj’. They also thought that it was a myth that water should be used on the burn as first-aid, instead, they used many other substances. Some examples of these are described below:

*“We usually remove the burnt skin; this is the death black skin, when we remove that fresh skin comes up, reddish. Then we put different things like toothpaste, egg, crushed potato, salty water, coconut oil, turmeric powder, or even red chili powder etc. Initially the person feels pain but in the long run, it makes a quick recovery”.*

Another participant felt that, *“Applying water to the burn injured place may cause further wound. Rather applying cow dung, kerosene or cumin oil, mud, blended banana are so useful. I come to know from my grandmother that applying cumin oil remove the pain and burning sensation.”* One of the male participants had a more accurate idea of what was beneficial for a burn: *“We use moist clay which makes immediate effect, it helps to cool the burn area. Sometimes we also put some water during applying clay”.*

### Health seeking behavior of rural communities

In the majority of cases, the participants reported that people seek health care from the local traditional healers and the Kabiraj. Village doctors and medicine shopkeepers were also commonly called upon, at home and work, in their role as health care providers, to see to burn injuries. The participants reported that the villagers were reluctant to go to the health care facilities, they think of them as expensive and that the quality of the service is not to their satisfaction. One participant was frustrated and mentioned that, due to formalities in the emergency department, it took an hour for their relative to be seen.

Participants also discussed the barriers to transporting patients to the nearby health facilities. One of the mothers gave the example of being dissatisfied with the care her child had received at a healthcare facility:

*“When my three-year child was burnt, immediately we went to the upazila health complex. They were not able to treat my child and referred to the district hospital. In the district hospital, I found their burn patient management negligent, they did not regularly do my child’s dressings”.*

Another respondent explained why they ended up using the Kabiraj for treating their relative:

*“My relative got burn and we went to the hospital, it was in Sadar (district) hospital. They provided initial treatment, bandage the wound and asked to immediately transfer the patient to Dhaka medical college burn unit. Dhaka is about six to seven hours distance from the district and we were not in a position to take my relative to Dhaka. We felt hopeless and returned back to our village, later our Kabiraj treated my relative. He is now much better”.*

The difficulty with access to transport was also highlighted by the participants especially transportation at night time.

*“It takes about one hour to bring the patient to the facility but the most difficult thing is to get transport to transfer the patient, especially when the burn occurs at night.”*

## Discussion

The provision of correct first-aid for a burn injury is a major challenge in rural Bangladesh. Rural populations in Bangladesh hold a number of misperceptions in relation to burn injuries and provide incorrect first-aid treatment and care. They have their own traditional beliefs and practices, the majority of which are harmful, for example, they believe pouring water on a burnt area is damaging. According to the participants, the incidence of burns is common in winter and the incidence is high among children and women with burns occurring most commonly in the cooking area. These findings are consistent with the findings of the Bangladesh National Health and Injury Survey [18, 19]. A high incidence of burn injuries is also observed in winter in the East Mediterranean Region: the incidence was higher among children and women and the home was found to be the most common place where burns happen [20, 21]. Using a cooker in an open area, carelessness during cooking, negligence in handling hot liquids and hot objects were identified in this study as being the main causes of burns. In a previous study undertaken in Bangladesh, an unsafe cooking environment, loose clothing and traditional open cookers have also been identified as risks for burns [18].

To change the villagers’ reluctance to treat a burn patient at the local health facility is a key challenge. In the case of a burn injury, the patient or family goes to the Kabiraj or traditional healers or they called the village doctors at home for treatment. This is not unique to the participants in this study, a previous study undertaken in Bangladesh found similar health seeking behavior for burn injuries: a reluctance to use the local health facilities [22].

Villagers who burn themselves, primarily depend on an unskilled person, like the *“Kabiraj” and traditional healer.* After that, the patient will then seek treatment from a qualified doctor at the upazila health complex or higher level health facilities. Most rural people are unwilling to receive treatment at government facilities in the upazila or the district because of their lack of knowledge, economic barriers and lack of the transport facilities to reach. Earlier studies undertaken in Bangladesh found that only about 20% of burn patients seek health care from government facilities [22]. They found that about 56% of victims took some action to treat the burn with 48.4% using medically unacceptable home remedies, for example, coconut oil, ghee, raw potato; only 5% were admitted to hospital [20].

In rural communities, there are many misperceptions about the correct treatment of burns. As stated above, some think that applying water is harmful for curing a burn, the majority believe that applying cool sand, cow dung, coconut oil, kerosene, mud and toothpaste on the wound enhances healing. Using local available substances on the burnt area is common practice and has been observed in other studies [23]. In our study we found people remove the skin of injured area immediately after a burn injury but this only makes the injury and the healing process more complicated.

Rural communities commonly use raw eggs, rotten parts of banana trees, cow dung and/or sand on the injured area. Some people soak the wounds with water repeatedly and apply ice to the wound to cool it down. First-aid practices for burn injuries people are adapted with pasting the area of the wound with mud, toothpaste, onion, raw potato mash, coconut oil, kerosene oil, and a mixture of limewater and coconut oil. A study in India has also found coconut oil and toothpaste used by rural communities for burn injuries [20]. Different herbal medicines are made from leaves of herbal plant, for example, sesame oil, sesame oil and wax combined or juice of ‘kapila’ leaves. Even a mixture prepared by boiling milk has been applied to the wound to avoid scarring. Communities also believe that applying heat to the burn will destroy the poison. These malpractices add to the complications that people with burns might experience [23, 24].

Several acute and chronic diseases may occur as a consequence of a burn injury. This includes skin and wound infections, gangrene, nervous disorder and even permanent disability. Annually in Bangladesh, about 2.6 per 100,000 population became permanently disabled [18]. The wrong treatment applied to burn injuries in rural communities in Bangladesh contribute to the harmful consequences of burn injuries.

The transportation of a burn injured patient to nearby upazila health complex is one problem highlighted by the participants in this study, as well as a dissatisfaction with the treatment received at that facility once the patient and their carers get there. A previous study found that about 60% of parents seek health care from an unqualified service provider for a child’s burn [25]. About 36% of people used harmful materials as a first aid measure for a burn injury; this led to detrimental consequences [26]. This may be a result of the lack of confidence that parents have in the service received at a health complex.

## Conclusions

Burns are one of the major causes of morbidity and disability among rural communities in Bangladesh, it appears that children and women are at the highest risk of burn injuries and that they most commonly occur in the kitchen area. This, together with the misperceptions and wrong treatment of burns, a reliance on traditional healers or unskilled staff highlight the need for community education programmes. Women and girls can be specifically targeted with simple burn prevention messages. In rural communities, school education about first-aid for burn injuries, as well as other common injuries, could be effective in reducing the incidence of burns and the severity of burns once they have occurred. Moreover, local government can play a pivotal role in translation of knowledge about burn prevention and immediate management to rural communities by using mass media. A wider community awareness campaign in rural settings will help to improve knowledge about burn injuries and the importance of immediate and correct treatment.

## Abbreviation

FGD: Focus group discussion
